# Deep Insight into the Influences of the Intrinsic Properties of Dielectric Elastomer on the Energy-Harvesting Performance of the Dielectric Elastomer Generator

**DOI:** 10.3390/polym13234202

**Published:** 2021-11-30

**Authors:** Yingjie Jiang, Yujia Li, Haibo Yang, Nanying Ning, Ming Tian, Liqun Zhang

**Affiliations:** 1 Beijing Advanced Innovation Center for Soft Matter Science and Engineering, Beijing University of Chemical Technology, Beijing 100029, China; jyj_940719@163.com (Y.J.); yujiali10930@163.com (Y.L.); yanghb@mail.buct.edu.cn (H.Y.); zhanglq@mail.buct.edu.cn (L.Z.); 2 Key Laboratory of Carbon Fiber and Functional Polymers, Ministry of Education, Beijing University of Chemical Technology, Beijing 100029, China

**Keywords:** dielectric elastomer, intrinsic property, energy harvesting

## Abstract

The dielectric elastomer (DE) generator (DEG), which can convert mechanical energy to electrical energy, has attracted considerable attention in the last decade. Currently, the energy-harvesting performances of the DEG still require improvement. One major reason is that the mechanical and electrical properties of DE materials are not well coordinated. To provide guidance for producing high-performance DE materials for the DEG, the relationship between the intrinsic properties of DE materials and the energy-harvesting performances of the DEG must be revealed. In this study, a simplified but validated electromechanical model based on an actual circuit is developed to study the relationship between the intrinsic properties of DE materials and the energy-harvesting performance. Experimental verification of the model is performed, and the results indicate the validity of the proposed model, which can well predict the energy-harvesting performances. The influences of six intrinsic properties of DE materials on energy-harvesting performances is systematically studied. The results indicate that a high breakdown field strength, low conductivity and high elasticity of DE materials are the prerequisites for obtaining high energy density and conversion efficiency. DE materials with high elongation at break, high permittivity and moderate modulus can further improve the energy density and conversion efficiency of the DEG. The ratio of permittivity and the modulus of the DE should be tailored to be moderate to optimize conversion efficiency (*η*) of the DEG because using DE with high permittivity but extremely low modulus may lead to a reduction in *η* due to the occurrence of premature “loss of tension”.

## 1. Introduction

The dielectric elastomer transducer (DET) has been a hot area of research in recent decades due to its high flexibility, light weight, large mechanical strain, simple structure and low cost [1,2,3,4]. A typical DET device consists of a dielectric elastomer (DE) film sandwiched by two compliant electrodes [5,6]. In generator mode, the DET is called the dielectric elastomer generator (DEG), and is able to convert mechanical energy into electrical energy during the stretch-release process due to its stretching variable capacitance property. As a new type of generator, the DEG provides a simple and feasible solution for harvesting energy from nature motion sources, such as waves, tides and human movements [7,8,9,10]. Therefore, the DEG has attracted much attention in recent years [11,12,13,14,15].

**Figure 1:**
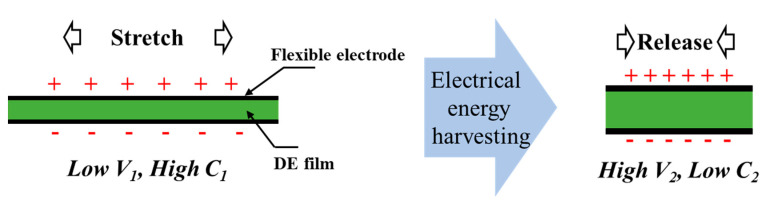
Schematic diagram of the working principle of the DEG. Mechanical energy is converted into electrical energy by releasing a stretched and charged DE film.

The working principle of the DEG is illustrated in Figure 1. The DE film is first electrically excited under low voltage at stretched state with high capacitance. Then, the film is released, and higher voltage across the film at the released state with lower capacitance can be obtained. The harvested electrical energy in this process is called the single-cycle generated energy (Δ*U*), which can be calculated as follows:

(1) $$\Delta U=U_{out}-U_{in}=\frac{1}{2}C_{2}V_{2}^{2}-\frac{1}{2}C_{1}V_{1}^{2}$$

Among them, *U_out_* and *U_in_* represent the input electrical energy in the stretched state and the output electric energy in the released state, respectively. *C*, *V* and the subscripts 1 and 2 represent the capacitance, voltage, the stretched state and the released state, respectively. The calculation formula of capacitance is:

(2) $$C=\frac{\varepsilon_{0}\varepsilon_{r}S}{z}$$

where *ε*_0_ represents the vacuum permittivity; and *ε_r_*, *S* and *z*, represent the relative permittivity (hereinafter referred to as permittivity), the area and the thickness of DE film, respectively. *C*, as well as Δ*U*, will be affected by the film size. Therefore, the gravimetric energy density (*w_m_*) and electromechanical conversion efficiency (*η*), as more important performances that avoid the influence of film shape [16], can be achieved by dividing Δ*U* by the mass of the DE film (*m*) and input mechanical work (*W_mech_*), respectively.

(3) $$w_{m}=\frac{\Delta U}{m}$$

(4) $$\eta =\frac{\Delta U}{W_{mech}}\times 100\%$$

Previous studies on the DEG have mainly focused on the circuit or device structure design to improve the energy-harvesting performance of the DEG based on commercial elastomers trademarked as VHB4905/10 [17,18,19,20,21,22,23]. On the other hand, some efforts have been made to prepare DE materials with high permittivity to enhance the performances of the DEG [24,25,26,27,28,29]. Ellingford et al. introduced a polar functional group into a styrene-butadiene triblock copolymer (SBS) to enhance the permittivity, but the significant charge leakage reduced the generated energy, which may have been caused by the relatively high conductivity of the modified SBS [28]. Yang et al. employed nature rubber (NR) and barium titanate (BT) as a high-elasticity DE matrix and dielectric filler, respectively, while the achieved conversion efficiency was relatively low, which may have resulted from the high modulus of the composite [26]. In these studies, the mechanical and electrical properties of the prepared materials were not well coordinated. Therefore, the energy-harvesting performances of the as-prepared DE materials were not satisfied. Therefore, to provide guidance for the preparation of high-performance DE materials for the DEG, the relationship between the intrinsic properties of DE materials and the energy-harvesting performances must be studied and revealed.

To date, scarce studies have reported the influences of the intrinsic properties of DE materials on the energy-harvesting performances of the DEG. Koh et al. established an electromechanical model to calculate the theoretical maximum energy density based on several failure mechanisms, and explored the influences of material parameters, such as Young’s modulus (*Y*), the product of permittivity and the square of electrical breakdown strength (*E_b_*), on the energy density [30]. This study provides a preliminary guidance for the preparation of high-performance DE materials for the DEG, but the results on energy density calculated by this model cannot be obtained in an actual circuit. Moreover, this model does not take the mechanics/charge loss of the DE into consideration. Therefore, the simulation results differ from the experimental results [22].

**Figure 2:**
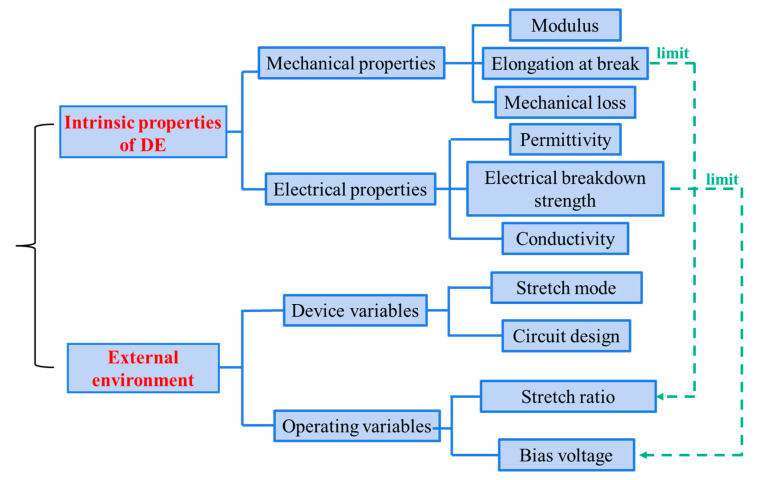
Influencing factors on the energy-generation performances of the DEG.

Therefore, an electromechanical model based on an actual circuit is necessary to reveal the relationship between the intrinsic properties of DE materials and the energy-harvesting performance, thereby guiding the design and preparation of DE. An electromechanical model would also help to predict the theoretical energy-harvesting performances of the DEG. Based on the energy generation mechanism of the DEG, the main influencing factors involved in the energy conversion are as follows. During the electrical excitation and harvesting process, the permittivity and strain, electrical breakdown strength and the bulk conductivity influence Δ*U* by affecting the capacitance, bias voltage and charge loss, respectively [31,32]. Therefore, the main influencing factors involved in energy conversion include the intrinsic properties of DE and the external environment, as summarized in Figure 2 [16,30]. Among these intrinsic properties, the mechanical-related properties include the Young’s modulus, elongation at break and mechanical loss, and the electrical related properties include the permittivity, electrical breakdown strength and bulk conductivity. The external environment factor can be divided into device variables, including the stretching mode and circuit design, and the operating variables, including stretch ratio and bias voltage. It is noted that some intrinsic properties of the material limit the maximum value of the operating variable. That is, the elongation at break and the electrical breakdown strength of the DE material limit the maximum stretch ratio and the maximum bias voltage that can be applied, respectively. Therefore, the energy-harvesting performance of the DE with different elongation at break and breakdown field strength can be equivalently investigated under different stretch ratios and bias voltages, respectively. Under the premise of fixed device variables, the relationship between the intrinsic properties of DE materials and the energy-harvesting performances can be studied.

In this study, a simplified but validated electromechanical model based on an actual circuit was developed to describe the relationship between intrinsic properties of DE material and the energy-harvesting performance. Linear elastic proposition with strain relaxation parameter was employed, and charge leakage was considered while studying the energy conversion mechanism. Experimental verification of the model was performed. The influences of six intrinsic properties of DE materials (including the modulus, elongation at break, mechanical loss property, permittivity, electrical breakdown strength and conductivity) on the energy-harvesting performances (including energy density and conversion efficiency) were systematically studied. Furthermore, guidance for the preparation of DE materials with high energy-harvesting performance was proposed. In addition, the ratio of permittivity and modulus of the DE material on the energy conversion efficiency of the DEG was discussed.

## 2. Modeling of DEG

### 2.1. Setup of Device Variables

**Figure 3:**
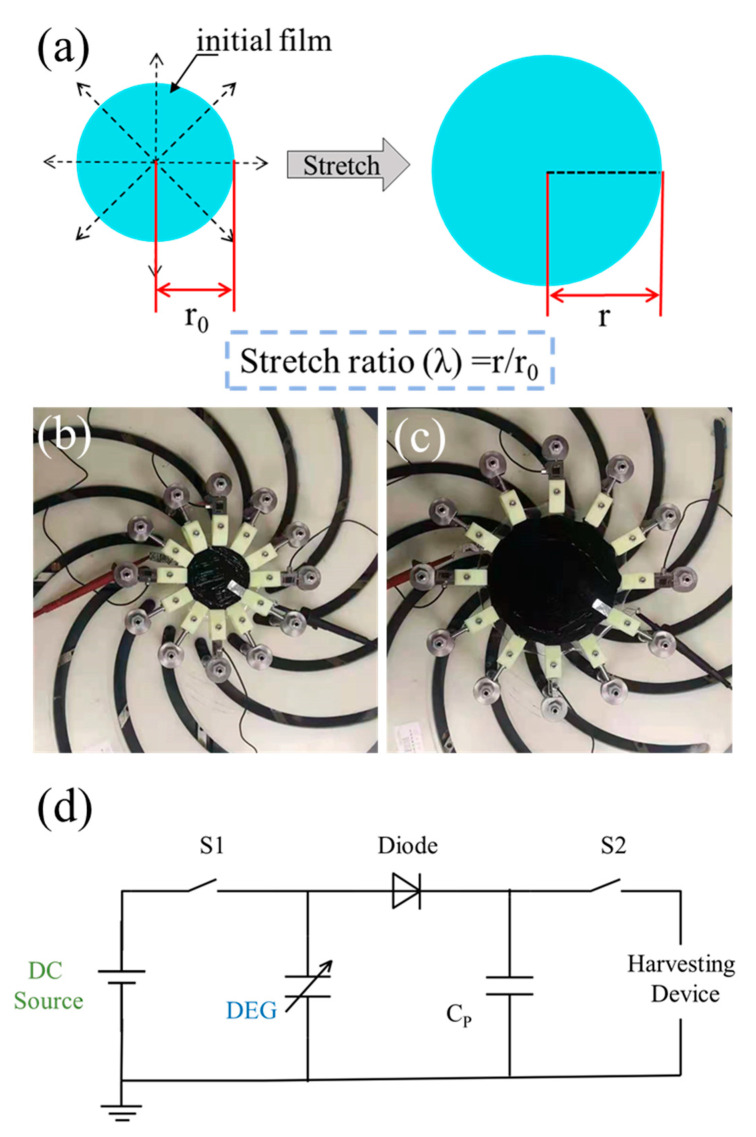
(**a**) Schematic diagram of equibiaxial stretch. (**b**) Released state and (**c**) stretched state of homemade equibiaxial stretch device. (**d**) Circuit principle adopted in this study.

The device variables contain the stretch mode and circuit design. The stretch modes reported in the literature have mainly included cone stretch, equibiaxial stretch and diaphragm inflatable stretch [21]. Among these stretch modes, equibiaxial stretch, a kind of uniform stretch in a plane, is the most widely used. This is because the uniformity of the thickness of the DE film can be maintained during stretching, and the highest energy-harvesting performances can be obtained under equibiaxial stretch [22,31]. Figure 3a shows a schematic diagram of equibiaxial stretch. The radius of the circular DE film before and after stretching are *r*_0_ and *r*, respectively. In this case, the equibiaxial stretch ratio *λ* is used to describe the degree of stretching, which is calculated using the formula *λ* = *r*/*r*_0_. The relationship between the stretch ratio and strain *ε* is *λ* = 1 + *ε*.

The circuit shown in Figure 3d was adopted according to Samuel Shian’s study [22]. The parallel transfer capacitors (*C_P_*) with the capacitance of *C_P_* = 1.3 *C_1_* in this circuit can reduce the voltage rise caused by film releasing to prevent the film from electrical breakdown during the releasing process and ensure the completion of the cycle. The DEG energy-harvesting process was performed through the following four steps: (i) the stretching process, where the equibiaxial stretch was performed on the DE film and changed its radius from *r*_0_ to *r*_1_; (ii) the voltage boosting process, where S1 was closed, and the DC source with preset input voltage *V*_1_ was made to fully charge the DE film and *C_P_*; (iii) the releasing process, where S1 was disconnected, and the DE film was released with a charged state, during which a higher voltage *V*_2_ can be obtained across the DE and *C_P_*. Because of the existence of Maxwell stress, a “loss of tension” occurred before it releasing to *r*_0_, and the radius of DE film at the released state was *r_k_* (*r*_k_ > *r*_0_); (iv) the harvesting process, where S2 was closed to release the charges across the DE film and *C_P_*.

The input mechanical work (*W_mech_*) is equal to the difference between the work done by the stretch device on the DE film (*W_s_*) during the stretch process and the work done by DE film on the stretching device (*W_r_*) during the release process, that is:

(5) $$W_{mech}=W_{s}-W_{r}=\int_{r_{0}}^{r_{1}}F_{s}dr-\int_{r_{k}}^{r_{1}}F_{r}dr$$

where *F_s_*, *F_r_* and *r*_1_ represent the stretching force, restoring force and the radius of stretched state, respectively.

**Figure 4:**
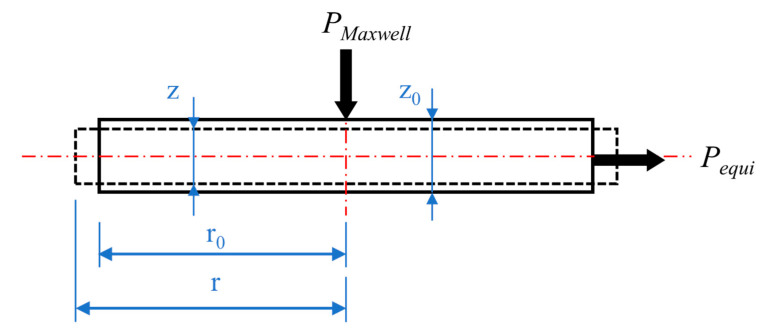
Schematic diagram of the Maxwell stress and equibiaxial stretch force acting on the DE film.

Both the equibiaxial stretch force and Maxwell stress perform work on the DE film during the energy-harvesting process. Figure 4 shows the schematic diagram of the Maxwell stress and equibiaxial stretch force acting on the DE film. Performing the Maxwell stress and equibiaxial stretch force from the thickness direction and horizontal direction on the DE film with an initial thickness of *z*_0_ and initial radius of *r*_0_, respectively, to make the film produce a slight deformation, the thickness becomes *z*_0_-*dz*, and the radius becomes *r*_0_ + *dr*.

Since the energy consumed by the two deformation methods is the same, the work performed by the Maxwell stress (*P_Maxwell_*) in the vertical direction is equal to the work performed by the equibiaxial stretch force (*P_equi_*) in the horizontal direction, that is:

(6) $$(P_{Maxwell}\pi r_{0}{}^{2})\cdot dz=(P_{equi}2\pi r_{0}\cdot z_{0})\cdot dr$$

Assuming that the volume of the DE film remains unchanged, that is:

(7) $$\pi r_{0}{}^{2}dz=2\pi r_{0}\cdot z_{0}dr$$

Combine the Equations (6) and (7):

(8) $$P_{Maxwell}=P_{\mathrm{equi}}$$

Therefore, the effect caused by *P_equi_* in horizontal direction is equivalent to that caused by the same magnitude of *P_Maxwell_* in the vertical direction.

During the stretching process, the stretch force applied on the circumference of the film causes the film to stretch in the radial direction and shrink in the thickness direction. At this time, the recovery force of the DE film is equal to its stretch force. Since the Maxwell stress also tends to shrink the film in the thickness direction and expand in the radial direction, the generation of the Maxwell stress caused by exerting bias voltage reduces the restoring force of the film. The input mechanical work is calculated based on the force-displacement relationship in the thickness direction. From Equation (8), during the releasing process of charged film, the restoring force is equal to equivalent *P_equi_* in the vertical direction minus *P_Maxwell_*. The value of *P_Maxwell_* under the action of the electric field strength (*E*) is *ε*_0_*ε_r_E*^2^. Therefore, during the releasing process of charged film, the vertical restoring force (*P_r_*) of the film under the action of the electric field is:

(9) $$P_{r}=P_{equi}-P_{Maxwell}=P_{equi}-\varepsilon_{0}\varepsilon_{r}E^{2}$$

Expressing the relationship between force and deformation in terms of Hooke’s law can simplify the model. Assuming that during the stretching process, the *P_equi_* and the stretch ratio *λ* satisfy the following linear relationship:

(10) $$P_{equi}=(\lambda -1)M$$

The proportional coefficient *M* is called the elastic coefficient. Since the effective modulus of equibiaxial stretching is twice of Young’s modulus [33], the relationship between *M* and Young’s modulus is *M* = 2 Y (1 + ε) = 2*λ*Y, where ε represents the strain. The higher Young’s modulus of the material results in the greater elastic coefficient *M*, so *M* can also reflect the ability of a material to resist elastic deformation under the action of external force.

### 2.2. The Description of Mechanical Loss Behavior

**Figure 5:**
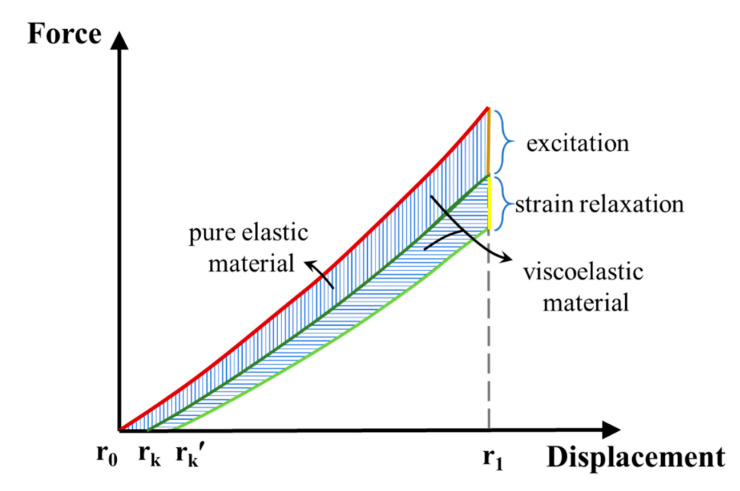
Force-displacement curve of the DEG under different elasticity conditions.

Rubber is a viscoelastic material [34]. During the stretch-release process, the movement of the molecular chain of the viscoelastic material needs to overcome the internal resistance to do work, and it must convert a part of the energy into heat energy, thus causing mechanical loss. After being stretched to a certain strain, the stress of rubber gradually decreases with time, a stress relaxation phenomenon [35,36]. During the voltage boosting process, it takes time for the voltage to increase from zero to the bias voltage value. Therefore, the DE film undergoes a stretching-relaxation-release process in the energy-harvesting cycle. The stress relaxation property is used in this work to describe the mechanical loss in the conversion process. The difference in the recovery force between the end of the stretching process and the beginning of releasing process comes from two aspects: One is the decrease caused by the Maxwell stress, and the other is the decrease caused by stress relaxation. In order to simplify the model description, the stress relaxation ratio *θ* was introduced to describe the mechanical loss behavior of the DE (i.e., the ratio between the relaxed stress and maximum stress) [37]. A smaller *θ* value indicates the better elasticity. At the beginning of the release process, the reduction of the recovery force caused by stress relaxation is directly deducted from the recovery force. DE is still regarded as a linear elastic material in the stretch and release process. In this study, the viscoelasticity of DE was considered, as shown in Figure 5. In this case, the restoring force of the film during the releasing process is as follows:

(11) $$P_{r}=P_{equi}-\theta \cdot P_{equi}(\lambda_{1})-P_{Maxwell}$$

where *P_equi_(λ)* represents the *P_equi_* under *λ*, and *λ*_1_ represents the stretched state ratio. In addition, *λ_k_* represents the released state ratio when the recovery force of the film drops to 0 and the film cannot continue to shrink. *λ_k_* can be calculated as follows:

(12) $$P_{equi}(\lambda_{k})-\theta \cdot P_{equi}(\lambda_{1})-P_{Maxwell}=0$$

### 2.3. The Description of Electrical Loss Behavior

Equation (14) describes the single-cycle generated energy without charge loss. However, the DE film is not an ideal insulator, so the charge will be consumed due to the tiny leakage current inside the DE. The leakage of charge is a continuous process which occurs from the charging of the DE film to the release of the charge.

**Figure 6:**
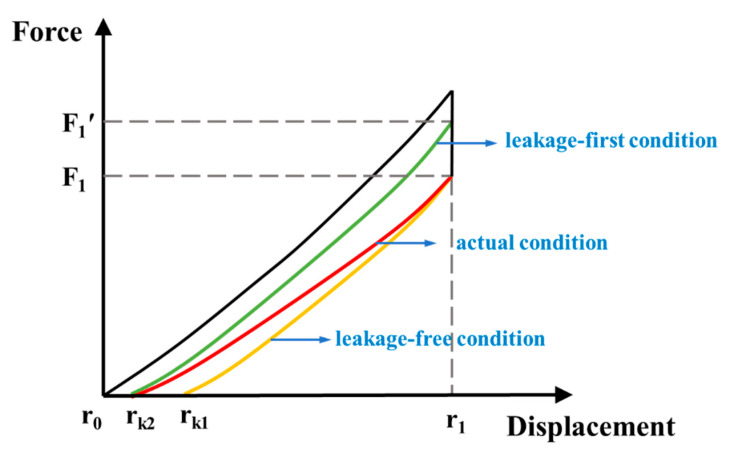
Force-displacement curve of the DEG under different charge leakage conditions.

Figure 6 shows the force-displacement curve under three different charge leakage conditions: (a) leakage-free condition, (b) actual condition and (c) leakage-first condition. The differences of these conditions are mainly exhibited in the release process. At the beginning of the release process (*λ*_1_, *F*_1_), since no charge has been consumed yet, the restoring force in the actual condition is equal to that in the leakage-free condition. During the release process, the continuous leakage of charge causes the voltage in the actual condition to be relatively lower than that in the leakage-free condition, that is, the Maxwell stress across the film during the actual condition is relatively low. Therefore, the restoring force in the actual condition is higher than that in the leakage-free condition. Therefore, the *r_k_* of the actual condition (marked as *r_k_*_2_) is smaller than the that of leakage-free condition (marked as *r_k_*_1_), that is, *r_k_*_2_ < *r_k_*_1_. Since the voltage and area of the film are constantly changing, other parameters, such as the stretching rate and time must be introduced in order to accurately express the charge leakage, which greatly complicates the model. To simplify the model, the charge leakage ratio *δ* is defined as the percentage of the leakage charge in the input charge. The higher conductivity of the DE material results in the higher leakage current. Therefore, *δ* is positively related to the conductivity of DE material, which is used to describe the electrical loss behavior of the material. *δ* can be calculated by the following formula:

(13) $$\delta =\frac{Q_{in}-Q_{out}}{Q_{in}}=\frac{(C_{1}+C_{P})V_{1}-(C_{2}+C_{P})V_{2}}{(C_{1}+C_{P})V_{1}}$$

At the end of the release process, the voltage across the DE film becomes:

(14) $$V_{2}=\frac{(C_{1}+C_{P})V_{1}\left(1-\delta \right)}{\left(C_{2}+C_{P}\right)}$$

Therefore, the actual single-cycle generated energy that takes the electrical loss into account can be rewritten from Equation (1) as:

(15) $$\Delta U=\frac{1}{2}\left(C_{1}+C_{P}\right)V_{1}^{2}\left[\frac{\left(C_{1}+C_{P}\right)}{\left(C_{2}+C_{P}\right)}{\left(1-\delta \right)}^{2}-1\right]$$

Under the definition of *δ*, the “leakage-first” condition is constructed, which means that the DE loses *δ* of the charge at the beginning of the process, and then the release process is performed with a constant charge. At the beginning of the release process, since no charge has been consumed yet in the actual condition, the voltage and the Maxwell stress in the actual condition are higher than that in the leakage-first condition, that is, *F*_1_ < *F*_1_*′*. As the film releases, the continuous leakage of the charge causes a decrease in the difference in the restoring force between the actual and leakage-first condition. Finally, when the restoring force in both cases drops to 0, the charge leakage ratio of both cases is *δ*, so the actual condition curve and the leakage-first condition curve intersect at the point (*r_k_*_2_, 0). Clearly, the release curve in the actual process is between that in the leakage-first condition and the leakage-free condition, so the average value of *W_r_* in the leakage-first condition and leakage-free condition can be used to approximate the *W_r_* in actual condition, which is:

(16) $$W_{r,actual}=\frac{W_{r,leakage-free}+W_{r,leakage-first}}{2}$$

### 2.4. The Model of Energy Harvesting Performances of DEG

By combining Equations (2) and (15), and Equations (5), (10), (12) and (16), the model on the energy-harvesting performances of the DEG can be obtained. Δ*U* and *W_mech_* can be written as:

(17) $$\Delta U=\frac{2.3\varepsilon_{0}\varepsilon_{r}\pi r_{0}^{2}\lambda_{1}^{4}V_{1}^{2}}{2z_{0}}\left[\frac{2.3\lambda_{1}^{4}}{1.3\lambda_{1}^{4}+\lambda_{k2}^{4}}\times {\left(1-\delta \right)}^{2}-1\right]$$

(18) $$\begin{array}{ll} W_{mech} & =2\pi r_{0}^{2}z_{0}M\left(\lambda_{1}-1-In\lambda_{1}\right)\\ \; & -\pi r_{0}^{2}z_{0}\left\{\begin{array}{l} \int_{\frac{1}{\lambda_{k1}^{2}}}^{\frac{1}{\lambda_{1}^{2}}}\left[\left(\lambda -1\right)M-\left(\lambda_{1}-1\right)M\theta -\frac{\varepsilon_{0}\varepsilon_{r}V_{1}^{2}}{z_{0}^{2}}\frac{{\left(2.3\lambda_{1}^{4}\right)}^{2}\lambda^{4}}{{\left(1.3\lambda_{1}^{4}+\lambda^{4}\right)}^{2}}\right]\lambda^{2}d\left(\frac{1}{\lambda^{2}}\right)\\ +\int_{\frac{1}{\lambda_{k2}^{2}}}^{\frac{1}{\lambda_{1}^{2}}}\left[\left(\lambda -1\right)M-\left(\lambda_{1}-1\right)M\theta -\frac{\varepsilon_{0}\varepsilon_{r}V_{1}^{2}{\left(1-\delta \right)}^{2}}{z_{0}^{2}}\frac{{\left(2.3\lambda_{1}^{4}\right)}^{2}\lambda^{4}}{{\left(1.3\lambda_{1}^{4}+\lambda^{4}\right)}^{2}}\right]\lambda^{2}d\left(\frac{1}{\lambda^{2}}\right) \end{array}\right\} \end{array}$$

where *r*_0_, *z*_0_, *M*, *θ*, *δ* and *ε_r_* represent the initial radius, initial thickness, elastic coefficient, stress relaxation ratio, charge leakage ratio and permittivity of DE film, respectively; and *V*_1_, *λ*_1_, *λ*, *λ_k_*_1_ and *λ_k_*_2_ represent bias voltage, stretch ratio, stretched state ratio and released state ratio without or with charge leakage, respectively. *λ_k_*_1_ and *λ_k_*_2_ can be obtained by:

(19) $$\left(\lambda_{k1}-1\right)M-\left(\lambda_{1}-1\right)M\theta =\frac{\varepsilon_{0}\varepsilon_{r}V_{1}^{2}}{z_{0}^{2}}\frac{{\left(2.3\lambda_{1}^{4}\right)}^{2}\lambda_{k1}{}^{4}}{{\left(1.3\lambda_{1}^{4}+\lambda_{k1}^{4}\right)}^{2}}$$

(20) $$\left(\lambda_{k2}-1\right)M-\left(\lambda_{1}-1\right)M\theta =\frac{\varepsilon_{0}\varepsilon_{r}V_{1}^{2}{\left(1-\delta \right)}^{2}}{z_{0}^{2}}\frac{{\left(2.3\lambda_{1}^{4}\right)}^{2}\lambda_{k2}{}^{4}}{{\left(1.3\lambda_{1}^{4}+\lambda_{k2}^{4}\right)}^{2}}$$

## 3. Results and Discussion

### 3.1. Experimental Validation

**Figure 7:**
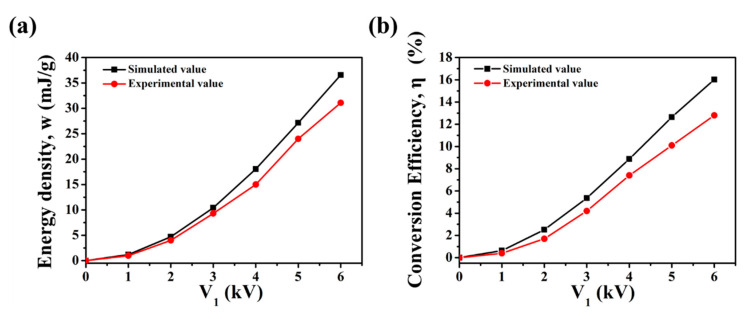
The experimental and simulated (**a**) energy density and (**b**) electromechanical conversion efficiency of the VHB4905 material under *λ*_1_ = 2 and different *V*_1_ values.

Before further exploration, the actual energy-harvesting performance of the VHB4905 material was measured under a homemade test platform in Section S1.3 in the Supporting Information and then compared with simulation results to verify the accuracy of the proposed model. The materials parameters used in the simulation were obtained by the characterization of VHB4905 (see Sections S2.1–S2.5 and Table S1 in Supporting Information). The experimental and simulated energy density and electromechanical conversion efficiency of the VHB4905 material under *λ*_1_ = 2 and different *V*_1_ values are shown in Figure 7. The results show that the error between the experimental value and simulated value of the energy density and conversion efficiency was less than 15% and 20%, respectively. These favorable results verify the feasibility of the proposed model in describing the relationship between the intrinsic properties of DE material and the energy-harvesting performances of the DEG. Moreover, the results indicate that the proposed model can predict the energy-harvesting performances of the DE.

It should be mentioned that the focus of this study is to establish the relationship between the intrinsic properties of materials and energy-harvesting performances, so some of the assumptions and approximations used in this model sacrifice accuracy. First, the linear elastic model with stress relaxation parameter is obviously different from the actual stretch-release process, so there is a certain error in the calculation of input mechanical work. Second, some studies have shown that the dielectric constant of the material changes with tension [38,39]. The dielectric constant was set as a constant in this model, which led to a certain error in the calculation of generated energy. The above factors will lead to an error between the model and the experimental value.

### 3.2. Influences of Intrinsic Properties of DE Materials on Energy-Harvesting Performance

The parameters related to the material properties in the model include the elastic coefficient M, stress relaxation ratio *θ*, permittivity *ε_r_*, bias voltage *V*_1_, stretched state ratio *λ*_1_ and charge leakage ratio *δ*. As explained in Section 2, the influences of the above six variables on the simulation results correspond to the influences of the modulus, viscoelasticity, permittivity, breakdown field strength, elongation at break and conductivity on the energy-harvesting performances, respectively. The six variables were divided into two groups: *V*_1_, *θ* and *δ* as one group, and permittivity *ε_r_*, elastic coefficient *M* and stretched state ratio *λ*_1_ as the other group. The influences of each variable on the energy-harvesting performances were studied separately.

To provide better guidance for preparing high-performance DE materials for the DEG, the values of these variables for the simulation were chosen according to experimental values. The shape of the DE film and the variables used in the simulation of energy-harvesting performances are shown in Table 1. Since the VHB material has atypical and serious viscoelasticity loss, the setting of *θ* value in this work refers to silicone rubber with high elasticity. The stress relaxation rate of the silicone rubber is around 0.05 to 0.10 [40]. Referring to this value, we set 0, 0.05 and 0.1, respectively, indicating no mechanical loss, high elastic material and moderate elastic material.

**Figure 8:**
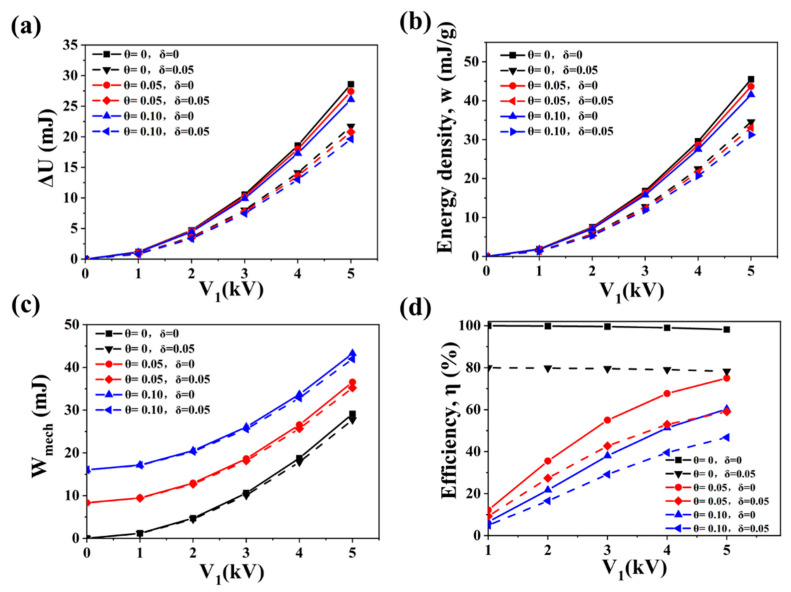
Influences of bias voltage *V*_1_, stress relaxation ratio *θ* and charge leakage ratio *δ* on the energy-harvesting performances of DE materials: (**a**) generated energy, (**b**) energy density, (**c**) input mechanical work, (**d**) electromechanical conversion efficiency. The abscissa represents the bias voltage; the black, blue and red curves represent the *θ* = 0, 0.05, 0.1, respectively; and the solid line and the dashed line represent the *δ* = 0 and 0.05, respectively.

The energy-harvesting performances of DE materials under different bias voltage *V*_1_, stress relaxation ratio *θ* and charge leakage ratio *δ*. The other three variables are fixed (elastic coefficient *M* = 0.2 MPa, permittivity *ε_r_* = 4.2, stretched state ratio *λ*_1_ = 2). Figure 8a shows the influences of the three variables above on the single-cycle generated energy Δ*U*. The increase in *V*_1_ greatly enhanced Δ*U*. The increase in *V*_1_ from 1 kV to 5 kV caused am increase in Δ*U* of 24 times. This is because the increase in *V*_1_ increased the work done by the Maxwell stress during the releasing process by enhancing the Maxwell stress. Thus, more mechanical energy can be converted into electrical energy. This also means that under the same film thickness, DE with higher breakdown strength can withstand a higher *V*_1_, which is able to obtain a higher Δ*U*. Δ*U* was significantly affected by charge leakage. Even a small charge leakage of 0.05 resulted in a significant decrease in Δ*U* of about 25%. Therefore, DE with lower conductivity can increase Δ*U* in the form of reducing charge leakage. The increase in *θ* increased the reduction of restoring force during the stress relaxation process, which increased *λ_k_* as well as *C*_2_, and thus decreased Δ*U*. Δ*U* was not sensitive to stress relaxation, but the effect of *θ* on Δ*U* increased with the increase in *V*_1_. In the case of *V*_1_ = 1 kV, *δ* = 0.05 and *θ* = 0.05, Δ*U* decreased by 2.9%. In the case of *V*_1_ = 5 kV, *δ* = 0.05 and *θ* = 0.05, Δ*U* decreased by 4.3%. Although Δ*U* was less affected by *θ*, DE with lower stress relaxation characteristics can obtain higher power generation, that is to say, high elasticity is needed for the ideal DE.

Figure 8b shows the influences of *V*_1_, *θ* and *δ* on the energy density *w*. The energy density was obtained by dividing Δ*U* by the mass of the DE in the effective working area, which reflected the energy-harvesting performance of DE per unit mass. The mass of the effective working area of DE remained unchanged under different variables. Therefore, the influences of the variables on the energy density were very similar to that on Δ*U*, except the scale of the ordinate was different. Similarly, the increase in *V*_1_, and the decrease of *δ* or *θ* all resulted in the increase in the energy density. Therefore, low conductivity, high breakdown strength and high elasticity are needed for DE materials with high energy density.

Figure 8c shows the variation of input mechanical work *W_mech_* with *V*_1_, *θ* and *δ*. The increase in *V*_1_ increases the *λ_k_* through enhancing the Maxwell stress, thus reduces the external work done by the film during the release process, which in turn leads to an increase in *W_mech_*. The increase in *θ* greatly increases the *W_mech_*, which reflects the energy loss caused by molecular chain rearrangement and slippage during the stretch-release process, and it is especially significant under low *V*_1_. In the case of low *V*_1_, the increase in *W_mech_* caused by *θ* is much higher than that caused by the *V*_1_. This means that most of *W_mech_* is consumed due to the viscoelastic loss, and only a small amount of *W_mech_* has been converted into electrical energy. The increase in *δ* slightly reduces the *W_mech_*. This is because the charge leakage reduces the work done by Maxwell stress, as analyzed in Section 2.3.

Figure 8d shows the influences of *V*_1_, *θ* and *δ* on the conversion efficiency *η*. The conversion efficiency was obtained by dividing Δ*U* by the input mechanical work *W_mech_*. *η* decreased with the increase in *δ*. Compared with the condition of *δ* = 0, *δ* = 0.05 will result in a decrease in *η* of nearly 20%. This is because the increase in *δ* greatly reduces Δ*U* but less affects *W_mech_*. Since the increase in *θ* had little effect on Δ*U* but largely increased *W_mech_*, *η* also decreased with the increase in *θ*. For example, under *V*_1_ = 1 kV and *δ* = 0.05, more *W_mech_* will be consumed in the viscoelastic loss by increasing *θ*, so the value of *η* drops from 79.97% (*θ* = 0) to 9.27% (*θ* = 0.05), and further reduces to 4.93% (*θ* = 0.1). This shows that using low elastic materials at low bias voltages is an inefficient way of harvesting energy. The change of *η* with *V*_1_ varied with the value of *θ*. In the case of *θ* = 0, no energy was consumed in the mechanical loss, and *η* decreased slightly with the increase in *V*_1_. In the case of *θ* ≠ 0, a large amount of the mechanical work was consumed in the mechanical loss, and the increase in *V*_1_ enlarged the Maxwell stress, thus increasing the proportion of the work done by the Maxwell stress to *W_mech_* during the conversion process, so the *η* was improved. The value of *η* gradually increased from 4.93% (*V*_1_ = 1 kV, *δ* = 0.05, *θ* = 0.1) to 46.79% (*V*_1_ = 5 kV, *δ* = 0.05, *θ* = 0.1). Therefore, the high breakdown strength, high elasticity and low conductivity of the material are of great significance for achieving high *η*.

**Figure 9:**
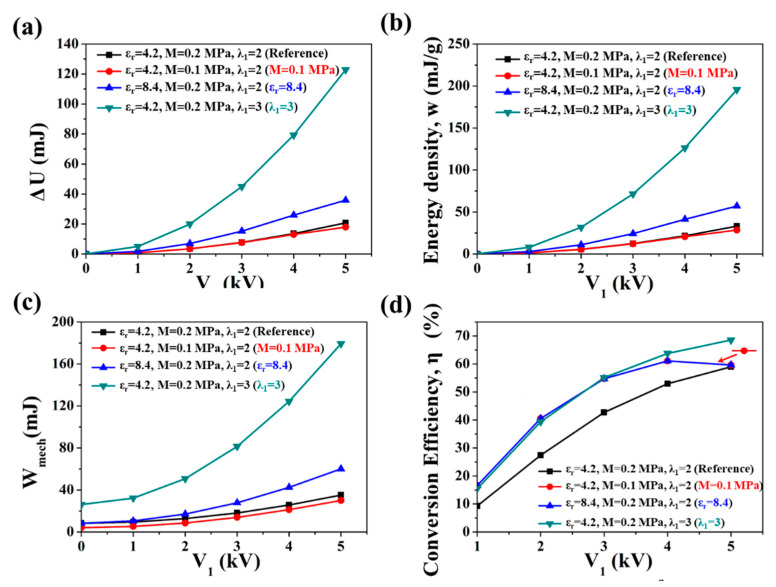
Influences of elastic coefficient M, permittivity *ε_r_* and stretched state ratio *λ*_1_ on the energy-harvesting performances of materials with under different *V*_1_: (**a**) generated energy, (**b**) energy density, (**c**) input mechanical work, (**d**) electromechanical conversion efficiency. The black “Reference” curve represents the material with fitting conditions of *ε_r_* = 4.2, M = 0.2 MPa, *λ*_1_ = 2; the red curve represents the material with fitting conditions of *ε_r_* = 4.2, M = 0.1 MPa, *λ*_1_ = 2; the blue curve represents the material with fitting conditions of *ε_r_* = 8.4, M = 0.2 MPa, *λ*_1_ = 2; the green curve represents the material with fitting conditions of *ε_r_* = 4.2, M = 0.2 MPa, *λ*_1_ = 3.

Figure 9 shows the energy-harvesting performances of DE materials with different elastic coefficient *M*, permittivity *ε_r_* and stretched state ratio *λ*_1_ under different *V*_1_ values. It is noticed that the black “Reference” curve represents the DE with the fitting conditions of *ε_r_* = 4.2, *M* = 0.2 MPa, *λ*_1_ = 2; the red “*M* = 0.1 MPa” curve represents the DE with the fitting conditions of *ε_r_* = 4.2, *M* = 0.1 MPa, *λ*_1_ = 2; the bule “*ε_r_* = 8.4” curve represents the DE with the fitting conditions of *ε_r_* = 8.4, *M* = 0.2 MPa, *λ*_1_ = 2; amd the green “*λ*_1_ = 3” curve represents the DE with the fitting conditions of *ε_r_* = 4.2, *M* = 0.2 MPa, *λ*_1_ = 3. The other two variables were fixed (*θ* = 0.05, *δ* = 0.05).

Figure 9a shows the influences of M, *ε_r_* and *λ*_1_ on Δ*U* under different *V*_1_ values. Since the increase in both *ε_r_* and *λ*_1_ increased the Maxwell stress, and the increase in *λ*_1_ also enlarged the stretch displacement, Δ*U* increased with the increase in *ε_r_*, and greatly increased with the increase in *λ*_1_. The simulation results show that under *V*_1_ = 5 kV, the increase in *ε_r_* to 2 times that of the reference sample caused the increase in Δ*U* from 20.77 mJ (Reference) to 35.84 mJ (*ε_r_* = 8.4), an increase of 72.6%. The increase in *λ*_1_ from 2 to 3 caused the increase in Δ*U* from 20.77 mJ (Reference) to 122.84 mJ (*λ*_1_ = 3), an increase of 491%. On the other hand, the decrease in M led to a slight decrease in Δ*U* under low *V*_1_, but when Δ*U* reached *V*_1_ = 5 kV, the decrease in M by half caused the decrease in Δ*U* from 20.77 mJ (Reference) to 17.92 mJ (*M* = 0.1 MPa), a decrease of 13.8%. This is because the decrease in M had no effect on Maxwell stress, but it reduced the recovery force, which increased *λ_k_* as well as the released state capacitance C_2_, thus reducing Δ*U*.

Figure 9b shows the influences of M, *ε_r_* and *λ*_1_ on the energy density *w* under different *V*_1_ values. Similar to Figure 8b, the influences of variables on *w* was very similar to that on Δ*U*, except that the scale of the ordinate was different. Therefore, the energy density increased with the increase in *ε_r_*, *λ*_1_ and *M*. The simulation results show that the material with the properties of *θ* = 0.05, *δ* = 0.05, *ε_r_* = 4.2, *M* = 0.2 MPa, and *λ*_1_ = 3 can obtain a theoretical energy density of up to 195.61 mJ/g under *V*_1_ = 5 kV.

Figure 9c shows the influences of *M*, *ε_r_* and *λ*_1_ on *W_mech_* under different *V*_1_ values. The simulation results indicate that *W_mech_* increased with the increase in *M*, *ε_r_* and *λ*_1_. The reduction in *M* reduced the useless work due to the viscoelastic loss, thus reducing *W_mech_*. For example, in the case of *V*_1_ = 5 kV, *W_mech_* reduced from 35.20 mJ (Reference) to 30.04 mJ (M = 0.1 MPa), a decrease of 14.7%. The increase in *ε_r_* indicatesan increase in Maxwell stress, which significantly increased *W_mech_*. Except for the increase in the work done by the Maxwell stress, the increase in *λ*_1_ also increased the mechanical loss, and thus largely increased *W_mech_*. In the case of *V*_1_ = 5 kV, the increase in *ε_r_* to 2 times that of the reference sample caused the increase in *W_mech_* from 35.20 mJ (Reference) to 60.08 mJ (*ε_r_* = 8.4), an increase of 70.7%. The increase in *λ*_1_ from 2 to 3 caused the increase in *W_mech_* from 35.20 mJ (Reference) to 179.20 mJ (*λ*_1_ = 3), an increase of 409%.

Figure 9d shows the influences of *M*, *ε_r_* and *λ*_1_ on *η* under different *V*_1_ values. Increasing *λ*_1_ can effectively improve *η,* especially under low *V*_1_. In the case of *V*_1_ = 1 kV, *η* increases from 9.27% (Reference) to 15.48% (*λ*_1_ = 3), an increase of 66.9%. In the case of *V*_1_ = 5 kV, *η* increases from 59.00% (Reference) to 68.55% (*λ*_1_ = 3), an increase of 16.2%. Interestingly, the blue and red curves almost overlapped, which shows that the influence of reducing *M* by half on *η* had the equivalent effect as that of increasing *ε_r_* by two-fold. The *M* and *ε_r_* values in the fitting condition of the blue curve (*M* = 0.2 MPa, *ε_r_* = 8.4) were twice those in red curve (*M* = 0.1 MPa, *ε_r_* = 4.2). Through extracting *M* or *ε_r_*, Equations (19) and (20) can be rewritten to Equations (21) and (22), respectively, from which *λ_k_*_1_ and *λ_k_*_2_ are equal in the two conditions under any *V*_1_ value.

(21) $$M\cdot \left[\left(\lambda_{k1}-1\right)-\left(\lambda_{1}-1\right)\theta \right]=\varepsilon_{r}\cdot \frac{\varepsilon_{0}V_{1}^{2}}{z_{0}^{2}}\frac{{\left(2.3\lambda_{1}^{4}\right)}^{2}\lambda_{k1}{}^{4}}{{\left(1.3\lambda_{1}^{4}+\lambda_{k1}^{4}\right)}^{2}}$$

(22) $$M\cdot \left[\left(\lambda_{k2}-1\right)-\left(\lambda_{1}-1\right)\theta \right]=\varepsilon_{r}\cdot \frac{\varepsilon_{0}V_{1}^{2}{\left(1-\delta \right)}^{2}}{z_{0}^{2}}\frac{{\left(2.3\lambda_{1}^{4}\right)}^{2}\lambda_{k2}{}^{4}}{{\left(1.3\lambda_{1}^{4}+\lambda_{k2}^{4}\right)}^{2}}$$

Similarly, through extracting *M* or *ε_r_*, Equations (17) and (18) can be rewritten as Equations (23) and (24), respectively.

(23) $$\Delta U=\varepsilon_{r}\cdot \frac{2.3\varepsilon_{0}\pi r_{0}^{2}\lambda_{1}^{4}V_{1}^{2}}{2z_{0}}\left[\frac{2.3\lambda_{1}^{4}}{1.3\lambda_{1}^{4}+\lambda_{k2}^{4}}\times {\left(1-\delta \right)}^{2}-1\right]$$

(24) $$\begin{array}{ll} W_{mech} & =M\pi r_{0}^{2}z_{0}\cdot \left\{2\left(\lambda_{1}-1-In\lambda_{1}\right)-\int_{\frac{1}{\lambda_{k1}^{2}}}^{\frac{1}{\lambda_{1}^{2}}}\left[\left(\lambda -1\right)-\left(\lambda_{1}-1\right)\theta \right]\lambda^{2}d\left(\frac{1}{\lambda^{2}}\right)-\int_{\frac{1}{\lambda_{k2}^{2}}}^{\frac{1}{\lambda_{1}^{2}}}\left[\left(\lambda -1\right)-\left(\lambda_{1}-1\right)\theta \right]\lambda^{2}d\left(\frac{1}{\lambda^{2}}\right)\right\}\\ \; & +\varepsilon_{r}\pi r_{0}^{2}z_{0}\left\{\int_{\frac{1}{\lambda_{k1}^{2}}}^{\frac{1}{\lambda_{1}^{2}}}\left[\frac{\varepsilon_{0}V_{1}^{2}}{z_{0}^{2}}\frac{{\left(2.3\lambda_{1}^{4}\right)}^{2}\lambda^{4}}{{\left(1.3\lambda_{1}^{4}+\lambda^{4}\right)}^{2}}\right]\lambda^{2}d\left(\frac{1}{\lambda^{2}}\right)+\int_{\frac{1}{\lambda_{k2}^{2}}}^{\frac{1}{\lambda_{1}^{2}}}\left[\frac{\varepsilon_{0}V_{1}^{2}{\left(1-\delta \right)}^{2}}{z_{0}^{2}}\frac{{\left(2.3\lambda_{1}^{4}\right)}^{2}\lambda^{4}}{{\left(1.3\lambda_{1}^{4}+\lambda^{4}\right)}^{2}}\right]\lambda^{2}d\left(\frac{1}{\lambda^{2}}\right)\right\} \end{array}$$

Therefore, the Δ*U* and *W_mech_* in the fitting condition of blue curve was twice of these in red curve under any *V*_1_, respectively. Thus, the value of *η* was the same under the two conditions. In this case, the two curves in the *η* graph vs. the *V*_1_ graph completely coincide. In addition, the *η* in these two fitting conditions first increased and then decreased with the increase in *V*_1_, reaching a maximum value of 61.11% at *V*_1_ = 4 kV. This indicates that DE with too high *ε_r_* or too low *M* is not conducive to obtain high *η* under high working voltage since a premature “loss of tension” may occur [31]. Therefore, it is necessary to balance the relationship between *E_b_* and *ε_r_* or *M* when designing high-*η* materials: Under low *E* (lower than 32 kV/mm), it is suggested to enhance *ε_r_* or reduce the modulus to improve *η*. Under high *E* (higher than 32 kV/mm), the ratio of *ε_r_* and *M* of the DE should be tailored to optimize *η*, and the recommended *ε_r_*/*M* value of the DE should be between 20/MPa and 40/MPa.

The results indicate that the Δ*U* and *w* were greatly affected by a small amount of charge leakage, but were not sensitive to mechanical loss property, so they could be significantly improved by increasing the insulation performance of the DE material. *W_mech_* was less affected by charge leakage but was quite sensitive to mechanical loss. In addition, Δ*U* and *w* can be further enhanced with the increase in *λ*_1_ and *ε_r_*. Appropriately reducing M is beneficial to improve *η*, but excessively low M will cause the reduction in *η* under high *V*_1_ since a premature “loss of tension” may occur. Since the reduction of the modulus and the increase in *ε_r_* have equivalent effects on *η*, DE materials with high *ε_r_* should have a moderate modulus. The recommended *ε_r_*/M value of DE should be between 20/MPa and 40/MPa.

To sum up, high breakdown field strength, low conductivity and high elasticity of DE materials are the prerequisites for obtaining high energy density and conversion efficiency. DE materials with high elongation at break, high permittivity and moderate modulus can further improve the energy density and conversion efficiency of the DEG.

## 4. Conclusions

Herein, an electromechanical model of DEG was established to reveal the relationship between the intrinsic properties of DE materials and energy-harvesting performances. The good agreement between the simulation and experimental results was verified, indicating that this coupling model can well predict the energy-harvesting performance of the material under the preset conditions. By tailoring the fitting condition in the model, the relationship between the intrinsic properties of DE materials (including the modulus, elongation at break, mechanical loss property, permittivity, breakdown field strength and conductivity) and the energy-harvesting performances (including the energy density and conversion efficiency) of the DEG was revealed. The results indicate that DE materials with high breakdown field strength, low conductivity and high elasticity are the prerequisites for achieving high energy density and high conversion efficiency of the DEG. In addition, DE materials with high elongation at break, high permittivity and moderate modulus can further improve the energy density and conversion efficiency of the DEG.

## Figures and Tables

**Table 1:** Summary of the shape of the DE film and variables in this study.

DE Film Shape and Variables	Value
thickness, *z*_0_/mm	0.5
radius, *r*_0_/mm	20
bias voltage, *V*_1_/kV	0, 1, 2, 3, 4, 5
stress relaxation ratio, *θ*	0, 0.05, 0.1
charge leakage ratio, *δ*	0, 0.05
elastic coefficient, *M*/MPa	0.2, 0.1
permittivity, *ε_r_*	4.2, 8.4
stretched state ratio, *λ*_1_	2, 3

## Data Availability

The data presented in this study are available on request from the corresponding author.

## Supplementary Materials

The following are available online at https://www.mdpi.com/article/10.3390/polym13234202/s1, Figure S1: Test platform contains equibiaxial stretching device, force/displacement sensor, digital electrometer, DC source and PC, Figure S2: Simulation of the elastic coefficient M of VHB4905 material. The slope of linear fitting curve of M = 0.2 MPa is adopted in simulation of the energy harvesting performances of VHB4905, Figure S3: The relationship between stress relaxation of VHB material and relaxation time under uniaxial strain of 100%. Stress relaxation ratio when time equal to 5s, that is θ = 0.35 is adopted in simulation of the energy harvesting performances of VHB4905, Figure S4: Permittivity vs. frequency graph of VHB4905. Permittivity under 100 Hz of ε_r_ = 4.2 is adopted in simulation of the energy harvesting performances of VHB4905, Figure S5: Calculated charge leakage ratio δ under different V_1_. Average value of δ is adopted in simulation of the energy harvesting performances of VHB4905, Table S1: Summary of value of DE film shape and variables in simulation of the energy harvesting performances of VHB4905.
