# Supplementary material for: Deep Insight into the Influences of the Intrinsic Properties of Dielectric Elastomer on the Energy-Harvesting Performance of the Dielectric Elastomer Generator
Source: Polymers (Basel). 2021 Nov 30;13(23):4202. doi: 10.3390/polym13234202 (PMC8659657; doi:10.3390/polym13234202)
Supplement: Supplementary file 1 [file polymers-13-04202-s001.zip › polymers-1464478-supplementary/Supporting Information.pdf]

Supporting Information

Deep Insight on the Influences of the Intrinsic Properties of Dielectric Elastomer on Energy Harvesting Performance of DEG

Yingjie Jiang[1], Yujia Li[1], Haibo Yang[1], Nanying Ning[1,2]*,

Ming Tian [1,2]* and Liqun Zhang[1,2]

1. Beijing Advanced Innovation Center for Soft Matter Science and Engineering, Beijing University of Chemical Technology, Beijing 100029, China;

2.Key Laboratory of Carbon Fiber and Functional Polymers, Ministry of Education, Beijing University of Chemical Technology, Beijing 100029, China;

*Corresponding authors. E-mail addresses: ningny@mail.buct.edu.cn (N. Y. Ning), tianm@mail.buct.edu.cn (M. Tian)

Part I: Experimental Section

1.1 Materials

Commercial polyacrylate elastomer film trademarked as VHB4905 is achieved from 3M Company, USA. Conductive carbon grease (GV-80S) used as compliant electrode is purchased from Yamate Co. Ltd, Japan. All chemicals are used as received.

1.2 Characterization

Permittivity (ε_r) of VHB4905 were measured by Broadband Dielectric Spectrometer (BDS, Concept 40, Novocontrol GmbH, German) over the frequency range of 10^{-1}-10^{6} Hz at room temperature. Mechanical properties of VHB4905 were investigated by using a universal mechanical testing machine (Shimadzu AG-IC, Japan) under the uniaxial tension mode. The circle specimens with the thickness of 0.5 mm and initial radius of r_0=20 mm was tested on the homemade equibiaxial stretching

device under a stretching rate of 300 mm/min until the break occurred. Five specimens were measured to confirm the stress−strain curves. Circle specimens with the same size were tested on the homemade equibiaxial stretching device under a stretching rate of 300 mm/min until reaching a strain of 100%, and the change of stretching force with relaxation time was recorded. Five specimens were measured to confirm the stress relaxation curves.

1.3 Measure method of energy harvesting performances

A platform based on the circuit principle shown in Figure 3b for testing energy generation performance of VHB4905 DEG has been completed (see the scheme in Figure S1) in this study. The experimental energy harvesting process was carried out through four subsequent steps: (i) stretching process, the circle VHB4905 film (DE film) with the thickness of 0.5 mm and initial radius of r_0=20 mm was first stretched under a constant radial speed of 300 mm/min by using a homemade equibiaxial stretching device, the radius of DE film changes from $r=r_0$ to $r=2r_0$, the strain is 100%;(ii) boosting process, connecting switch 1 to fully charge the DEG and C_P for 5 s; (iii) release process, disconnecting switch 1 and releasing the DEG under a speed of 600 mm/min, the radius of DE film changes from $r=2r_0$ to $r=1.25r_0$; and (iv) harvesting process, connecting switch 2 to measure the electrical energy stored in the DEG.

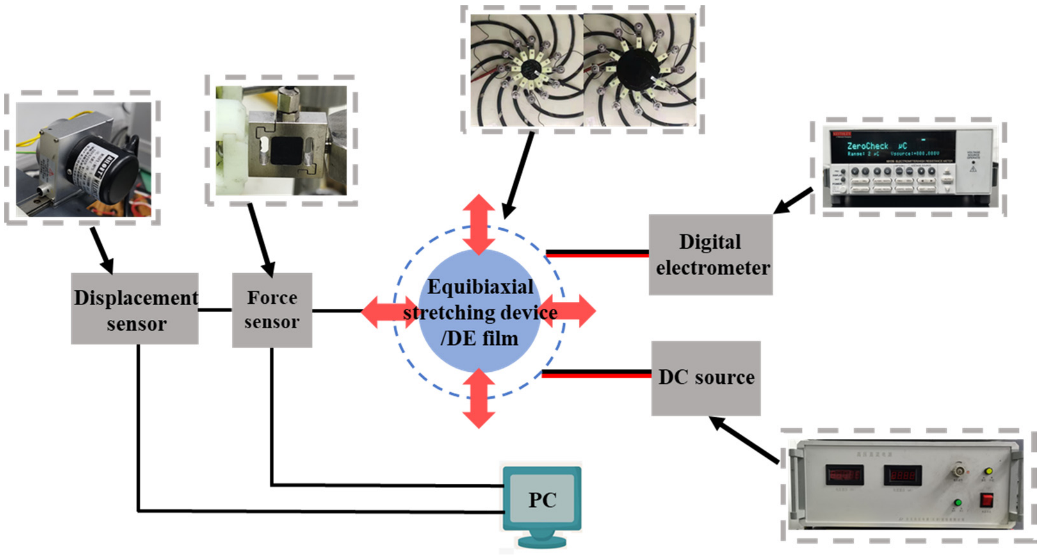

Figure S1. Test platform contains equibiaxial stretching device, force/displacement sensor, digital electrometer, DC source and PC.

The input bias voltage (V_1) was controlled by a high voltage DC source (Model DW-P803, Dongwen HV Source Co. Ltd.), the capacitance of elastomers at stretched state (C_1) and the capacitance of elastomers at released state (C_2) were measured by using a digital electrometer (Model 6517b, Tektronix Co. Ltd.). The measured voltage of the DEG at relaxed state (V_2') was recorded by the same digital electrometer. Note that a high-voltage dividers ($R_{divider}$ ~ 50 GΩ) were used to help measure the high voltages with a low charge loss. The experimental generated energy ΔU can be calculated by following:

$$\Delta U = U_{out} - U_{in} = \frac{1}{2}\left[\left(C_2 + C_P\right)V_2'^2 - \left(C_1 + C_P\right)V_1^2\right] \quad \text{(S1)}$$

The energy density w can be achieved by dividing the generated electric energy by the input mechanical energy The force-displacement relationship was recorded by the force sensor and displacement sensor, so that input mechanical work W_{mech} can be calculated. Then the electromechanical conversion efficiency η can be further obtained by dividing the ΔU by W_{mech}.

Part II: Material parameters of VHB4905

2.1 Stretched state ratio λ_1 and bias voltage V_1

λ_1=2 or strain of 100% is adopted.

V_1=1~6 kV with an increment of 1 kV are adopted.

2.2 Elastic coefficient M

According to Equation (10), elastic coefficient M can be obtained as the slope of pressure and strain. Therefore, the slope of the linear fitting results of the experimental ture stress-strain curve under uniaxial stretching within strain under 100%, that is, M=0.2 MPa is adopted in simulation of the energy harvesting performances of VHB4905, as shown in Figure S2.

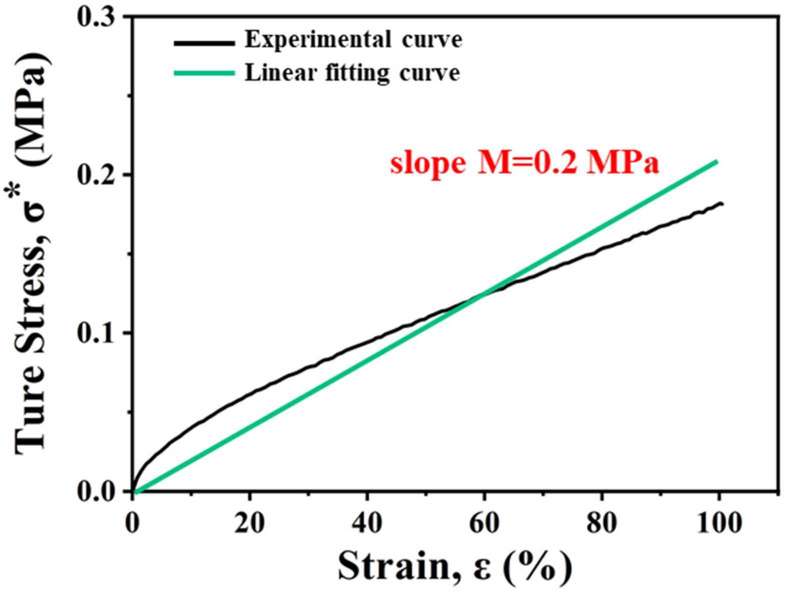

Figure S2. Simulation of the elastic coefficient M of VHB4905 material. The slope of linear fitting curve of M=0.2 MPa is adopted in simulation of the energy harvesting performances of VHB4905.

2.3 Stress relaxation ratio θ

During the boosting process, it takes 5s to fully charge the DE film while maintain the stretching state of DE. And the stress relaxation of DE film occurs in this process. Therefore, stress relaxation ratio when time equal to 5s, that is θ=0.35

is adopted in simulation of the energy harvesting performances of VHB4905, as shown in Figure S3.

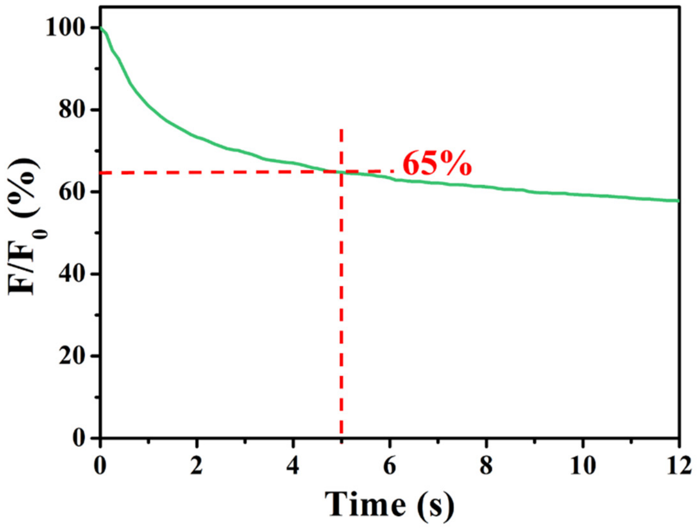

Figure S3. The relationship between stress relaxation of VHB material and relaxation time under uniaxial strain of 100%. Stress relaxation ratio when time equal to 5s, that is θ=0.35 is adopted in simulation of the energy harvesting performances of VHB4905.

2.4 Permittivity ε_r

Since the charging of DEG is a direct-current process,[1] the ε_r at the lowest frequency (10^0 Hz) of VHB4905, that is ε_r=4.2 is adopted in simulation of the energy harvesting performances of VHB4905, as shown in Figure S4.

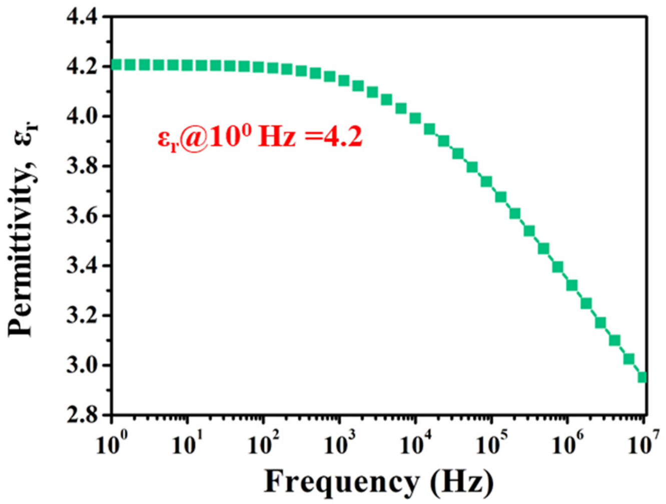

Figure S4. Permittivity vs frequency graph of VHB4905. Permittivity under 10^0 Hz of ε_r= 4.2 is adopted in simulation of the energy harvesting performances of VHB4905.

2.5 Charge leakage ratio ω

The charge leakage ratio under different V_1 can be calculated by Equation (12), average value of ω=0.03 is adopted in simulation of the energy harvesting performances of VHB4905.

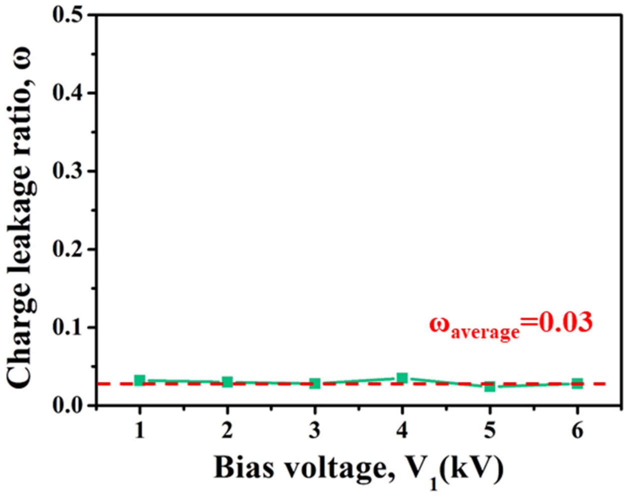

Figure S5. Calculated charge leakage ratio ω under different V_1. Average value of ω is adopted in simulation of the energy harvesting performances of VHB4905.

Table S1 Summary of value of DE film shape and variables in simulation of the energy harvesting performances of VHB4905.

DE film shape	Value
thickness, z_0/mm	0.5
radius, r_0/mm	20
**Variables**	**Value(s)**
bias voltage, V_1 /kV	1, 2, 3, 4, 5, 6
strain relaxation ratio, θ	0.35
charge leakage ratio, ω	0.03
elastic coefficient, M/ MPa	0.2
permittivity, ε_r	4.2
stretched state ratio, λ_1	2

1. Shian, S.; Huang, J.; Zhu, S.; Clarke, D. R., Optimizing the electrical energy conversion cycle of dielectric elastomer generators. *Advanced Materials* **2014,** *26* (38), 6617-6621.
